# Physical activity practice and sports preferences in a group of Spanish schoolchildren depending on sex and parental care: a gender perspective

**DOI:** 10.1186/s12887-020-02229-z

**Published:** 2020-07-07

**Authors:** África Peral-Suárez, Esther Cuadrado-Soto, José Miguel Perea, Beatriz Navia, Ana M. López-Sobaler, Rosa M. Ortega

**Affiliations:** 1 Department of Nutrition and Food Science, Faculty of Pharmacy at Universidad Complutense de Madrid, Madrid, Spain; 2 Department of Nutrition, Faculty of Health Science at Universidad Alfonso X El Sabio, Villanueva de la Cañada, Madrid, Spain; 3 Research Group VALORNUT-UCM (920030), Universidad Complutense de Madrid, Madrid, Spain

**Keywords:** Physical activity, Sport preferences, Schoolchildren, Sex differences, Gender, Parental influence

## Abstract

**Background:**

Physical activity plays an important role in the maintenance of health, and it is especially important during childhood. However, the lack of information about differences in physical activity practice and sports preferences of children considering gender differences can result in non-effective policies that enhance inequalities between sexes. The aim of this study is to identify the sports preferences of Spanish schoolchildren and their physical activity practice behaviors depending on their sex and their parental care, analyzing the possible differences from a gender perspective.

**Method:**

Three hundred sixty-four Spanish schoolchildren (179 girls, 185 boys) participated in this cross-sectional study. A daily physical activity questionnaire was used to evaluate physical activity level (PAL), moderate-to-vigorous physical activity (MVPA) and sports preferences and a socio-health questionnaire were used to collect data about parental care. Statistical analysis was performed using SPSS and applying Student’s T-test for normal variables, Mann-Whitney U-test for non-parametrical variables, and chi-square (χ2) test for categorical variables. Subsequently, odds ratios were used to analyze associations between the physical activity practice of the children and parental care.

**Results:**

PAL and time spent in MVPA was significantly lower for girls compared to boys (1.44 ± 0.07 vs. 1.46 ± 0.07, *p* < 0.001 and 0.74 ± 0.40 h/day vs. 0.90 ± 0.45 h/day; *p* < 0.001, respectively). Dancing, rhythmic gymnastics, skating, and water sports were practiced more by girls, while football, wrestling sports, handball, and racket sports were practiced more by boys (*p* < 0.05). Children cared for by their fathers had higher odds for physical activity practice (OR = 1.995 (1.202–3.310), *p* = 0.008).

**Conclusion:**

Physical activity among girls was less frequent and less intense. Girls opted for individual sports with artistic connotations, while boys often practiced more team contact sports. Furthermore, children are more physically actives when their father is in charge of them.

## Introduction

Regular physical activity has numerous benefits for both physical and mental health, including the prevention of being overweight or obese, and the risk of chronic diseases (cardiovascular, diabetes, cancer, etc.), as well as reduced levels of stress and anxiety, impacting on psychological wellbeing and quality of life [1, 2]. Furthermore, in the case of schoolchildren, physical activity has been positively associated with academic performance [3]. Physical inactivity is related to greater risk of diseases and various physiological and psychological problems, being identified as the fourth highest risk factor for mortality worldwide [1, 4].

In this context, physical activity and sports practice tend to be different between boys and girls, being lower in the case of girls, both in terms of frequency and intensity, independent of their level of education [5, 6].

Among possible barriers that girls face when engaging in physical activity and sports are the gender stereotypes associated with physical activity due to the masculine image it projects. This can lead to a refusal to participate in “male sports” for parts of the female population [7, 8]. Increased difficulty achieving required goals generates huge pressure for females [9]. These gender roles are assumed from an early age [7].

The results from various past studies show that girls tend to prefer activities related to body shape and health with a more aesthetic orientation, preferring individual sports, while boys tend to opt for activities focused on improving fitness or physical performance, choosing team sports in which strength and competitiveness predominate [10, 11].

Several studies have shown that practice of physical activity by parents or parental support in this area may improve physical activity by the children. However, the difference between maternal and paternal influence is not clear. In some studies, it was observed that both figures have equal influence. While in others, it was indicated that the figure that shares the sex of the schoolchild has more influence, or that the paternal figure is the most influential [12–15].

Most research on the practice of physical activity by schoolchildren focuses on time or on active transportation, ignoring the preferences of children for particular sports or types of activity. Researching these factors could improve our understanding of the reasons why they enjoy said preferences [16].

The aim of this study was to determine the differences in sports preferences of schoolchildren, as well as their practice of physical activity depending on their sex, in addition to investigating the influence children’s parents or guardians in their activeness. We analyze all these factors from a gender perspective.

## Materials and methods

The study design and methodology have been previously described [17–19]. The study was conducted in accordance with the Declaration of Helsinki, and the protocol was approved by the Ethics Committee for Clinic Review of the Clinic San Carlos Hospital, which is part of the Universidad Complutense de Madrid (Madrid, Spain) (Ref 12/319-E and 15/522-E). The trial was registered at clinicaltrials.gov as NCT03465657.

In brief, a cross-sectional observational study was carried out between February 2014 and February 2018 in 11 randomly contacted schools from different Spanish provinces, with representation from urban and semi-urban areas, in which 367 participated healthy children aged between 7 and 11 years. The obtaining of the sample and methodology used to collect the different data is deeply described below.

### Subjects

**Fig. 1:**
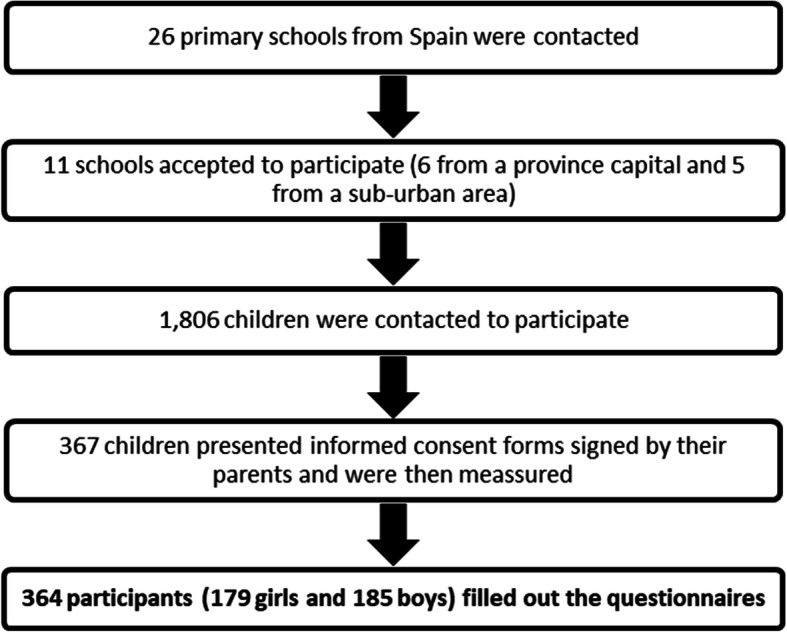
Obtaining the sample

Participation in this study was offered to twenty-six randomly-contacted primary schools. Eleven schools from six different Spanish provinces (Madrid, Zaragoza, Segovia, Córdoba, Ciudad Real and Tenerife) took part in the project, of which five were in the capitals of the provinces involved and six in a semi-urban area (less than 50,000 inhabitants). Of the 1806 children who were contacted to participate, a sample of 367 children was recruited to the trial, (182 girls, 185 boys) (Fig. 1).

Each school was contacted by telephone. Once the schools Directors had accepted the invitation to participate, parents of children aged between 7 and 11 years received a letter explaining the study in detail and were given an informed consent form to fill out, as to whether they agreed with the participation of their child. Afterwards, a member of the research group met the parents who accepted the participation of their children in the study at their child’s schools. This was to discuss any doubt about the trial and collect the signed informed consent forms. In said initial meeting, the member of the research group provides parents with the questionnaires to complete. Five days after the meeting, the researchers went to the school to carry out the anthropometric measurements and collect completed questionnaires.

The exclusion criteria were: a lack of signed informed consent, having an illness or physical impairment that could alter the results (serious infection or metabolic or chronic diseases as diabetes mellitus, hepatic or kidney disease), having have had surgery in the 6 months prior to the study and lack of completed questionnaires.

Children whose questionnaires had missing answers for isolated questions were excluded from the analysis of those particular questions but not from the whole study.

### Anthropometric and Sociodemographic data

All anthropometric measurements were taken in the morning and in accordance with the WHO criteria [20], namely the children being barefoot and wearing just their underwear. The children entered in small groups of approximately 5 children into the room where the measurements were being taken and were measured one by one in a space separated from the rest of the room by folding screens by two researchers. One of the researchers was in charge of taking the measurements and the other was in charge of noting the results.

Data about weight and height were determined using a digital electronic scale (range 0.1–150 kg; precision 100 g; Alpha; Seca, Igni, France) and a digital stadiometer (70–205 cm; 1 mm; Harpenden Pfifter, Carlstadt, NJ, USA) respectively. The body mass index (BMI) was calculated from these measures.

Weight status was determined using BMI specific percentiles for age and sex in the reference population following the criteria stablished by the International Obesity Task Force (IOTF) [21].

Waist circumference was measured using a flexible metallic tape (range 0–150 cm; precision 1 mm; Holtain, Crymych, Wales). The measurement was taken midway between the inferior margin of the last rib and the crest of the ileum, in the horizontal plane.

To obtain the sociodemographic data, we used a questionnaire about their social, economic, and health status [see Additional File 1], which were completed by their parents. This questionnaire included data on the children’s caregivers, including the academic level of their parents and the household incomes.

### Physical activity data

An adapted physical activity daily questionnaire [22] [see Additional File 2], which has been previously used in other studies [23–25], was filled out by the parents about their children. Questions find the time spent during weekdays and weekends in different kind of physical activities (including active play and extracurricular sport classes, physical education and daily life activities) and sedentary behaviors (including watching TV, playing videogames, tablet or computer use). Furthermore, questions were included about the kinds of sports practiced during extracurricular sporting classes, including the number of days per week and the time per session dedicated to each extracurricular sport class.

After collecting the information, individual physical activity levels (PAL) were calculated by multiplying the time in hours spent on each group of activities with their assigned coefficient depending on their intensity (1.0 for rest, 1.5 for very low-intensity PA, 2.5 for low-intensity PA, 5.0 for moderate-intensity PA and 7.0 for very high-intensity PA) following the WHO criteria [26]. Additionally, the reported mean of moderate to vigorous physical activity (MVPA) hours per day was quantified, considering those activities with the assigned factors 5 or 7 [26], which included: physical education in school and extracurricular physical activities. The reported mean of sedentary leisure hours per week was computed, considering the use of electronic displays (computer, videogame console, tablet, and TV). This data was used to estimate adherence to the recommendations of physical activity (≥60 min MVPA per day) and screen time (≤2 h per day) contained in the guidelines [27, 28].

We also classified children as non-sedentary when their PAL was 1.4 or higher [29].

### Statistical analysis

All the results were analyzed using the IBM SPSS Inc. statistical software (version 25.0) and they were shown as mean ± standard deviation (SD), medians, and interquartile range (IQR) or as proportions if variables were categorical. The Kolmogorov–Smirnov test was used to assess the normality of the variables. To compare data between sexes, we used the Student’s two sample t-test (in cases of normal variables), Mann–Whitney U-test (in cases of non-parametrical variables), and χ2 test for categorical variables.

Subsequently, logistic regression models were used to analyze the possible association between physical activity of the children (dependent variable) and the person who takes care of them (independent variables), using as reference for physical activity practice: PAL ≥1.4, according to National Academy of Medicine (NAM) cut-off points, previously known as Institute of Medicine (IOM) [29]. This association was evaluated by 3 models of the odds ratio (OR) using 95% confidence intervals (CI): (a) a basic model without any adjustment, (b) a second model taking into account sex and age, and (c) a third model including model b plus the rest of the predictor variables: cared by mother (yes/no), cared by father (yes/no) or cared by others (yes/no).

Differences were considered statistically significant if the *p*-value < 0.05.

## Results

From the initial sample of 367 children (182 girls and 185 boys), three girls did not answer at least one of the questionnaires, so the final sample was reduced to 364 children (179 girls and 185 boys).

The mean age of the participants was 8.98 ± 1.21 years. No significant differences between sexes were found analyzing anthropometric measures and sociodemographic data collected in Table 1.

**Table 1:** Anthropometric and sociodemographic data of the study sample according to sex

	Girls (***n*** = 179)	Boys (***n*** = 185)	Total (***n*** = 364)	
**Anthropometric data**				
	**Mean ± SD**	**Mean ± SD**	**Mean ± SD**	***p***
**Age (years) (†)**	8.98 ± 1.23	8.98 ± 1.20	8.98 ± 1.21	0.916
**Weight (kg) (†)**	35.89 ± 9.04	36.00 ± 8.53	35.95 ± 8.77	0.908
**Height (cm)**	136.87 ± 9.49	137.89 ± 8.29	137.39 ± 8.90	0.276
**BMI (kg/m**^**2**^**) (†)**	18.93 ± 3.27	18.78 ± 3.34	18.85 ± 3.30	0.434
**Waist circumference (cm) (†)**	63.77 ± 8.18	64.59 ± 9.52	64.19 ± 8.88	0.674
**Weight status**				
	***n*(%)**	***n*(%)**	***n*(%)**	***p***
**Classification BMI (IOTF)**				0.377
Underweight	10 (5.59)	5 (2.70)	15 (4.12)	
Normal weight	98 (54.75)	112 (60.54)	210 (57.69)	
Overweight	54 (30.17)	48 (25.95)	102 (28.02)	
Obesity	17 (9.50)	20 (10.81)	37 (10.16)	
**Sociodemographic data**				
	***n*(%)**	***n*(%)**	***n*(%)**	***p***
**Academic level of father**				0.200
No academic education	3 (1.79)	2 (1.12)	5 (1.45)	
Primary school	41 (24.40)	36 (20.22)	77 (22.25)	
High school/VT	61 (36.31)	86 (48.31)	147 (42.49)	
University degree	56 (33.33)	45 (25.28)	101 (29.19)	
Master/PhD	6 (3.57)	9 (5.06)	15 (4.34)	
**Academic level of mother**				0.490
No academic education	0 (0.00)	0 (0.00)	0 (0.00)	
Primary school	34 (19.10)	30 (16.57)	64 (17.83)	
High school/VT	66 (37.08)	79 (43.65)	145 (40.39)	
University degree	68 (38.20)	66 (36.46)	134 (37.33)	
Master/PhD	10 (5.62)	6 (3.31)	16 (4.46)	
**Household incomes**				0.225
Less than 12,000 €/ year	18 (11.8)	20 (12.7)	38 (12.3)	
12,000€-30,000€ / year	56 (36.6)	74 (47.1)	130 (41.9)	
30,001€-48,000€ / year	42 (27.5)	33 (21.0)	75 (24.2)	
More than 48,000€/ year	37 (24.2)	30 (19.1)	67 (21.6)	
**Habitat**				0.734
Province capital	98 (54.75)	98 (52.97)	196 (53.85)	
Semi-urban area	81 (45.25)	87 (47.03)	168 (46.15)	
**Children care**				0.088
Mother	60 (33.52)	62 (33.88)	122 (33.70)	
Father	4 (2.23)	7 (3.83)	11 (3.04)	
Another person	10 (5.59)	7 (3.83)	17 (4.70)	
Mother + Father	59 (32.96)	81 (44.26)	140 (38.67)	
Mother + Other	21 (11.73)	14 (7.65)	35 (9.67)	
Father + Other	1 (0.56)	1 (0.55)	2 (0.55)	
Mother + Father + Other	24 (13.41)	11 (6.01)	35 (9.67)	

*SD* Standard deviation; *BMI* Body mass index; *VT* Vocational Training. †: *p*-value calculated by Mann–Whitney U-test

### Physical activity and sedentary behavior according to sex

The results of Table 2 show that boys performed more (*p* < 0.001) and more intense (*p <* 0.001) physical activity than girls, spending more time on attending extracurricular sport classes (*p* = 0.001) and on active playing (*p* = 0.014).

**Table 2:** Differences in physical activity practice based on sex

	Girls (***n*** = 179)		Boys (***n*** = 181)		Total (***n*** = 360)		
Physical activity and sedentary behavior indicators							
	Mean ± SD	Median	Mean ± SD	Median	Mean ± SD	Median	***p***
		(IQR)		(IQR)		(IQR)	
PAL (†)	1.44 ± 0.07	1.43	1.46 ± 0.07	1.46	1.45 ± 0.07	1.44	**< 0.001**
		(1.39–1.48)		(1.41–1.51)		(1.40–1.50)	
MVPA (h/day) (†)	0.74 ± 0.40	0.70	0.90 ± 0.45	0.86	0.82 ± 0.43	0.71	**< 0.001**
		(0.43–0.94)		(0.57–1.14)		(0.52–1.07)	
Attendance at extracurricular sport classes (days/week) (†)	1.8 ± 1.4	2.0	2.7 ± 1.6	3.0	2.3 ± 1.6	2.0	**< 0.001**
		(1.0–3.0)		(2.0–4.0)		(1.0–3.0)	
Time spent in extracurricular sport classes (h/day) (†)	0.38 ± 0.34	0.29	0.50 ± 0.36	0.43	0.44 ± 0.35	0.36	**0.001**
		(0.14–0.57)		(0.29–0.71)		(0.21–0.64)	
Active play (h/day)	1.48 ± 1.02	1.29	1.69 ± 0.98	1.64	1.59 ± 1.00	1.57	**0.014**
		(0.71–2.07)		(1.00–2.29)		(0.91–2.25)	
Use of PC/console/tablet (h/day) (†)	0.76 ± 0.58	0.64	1.01 ± 0.78	0.86	0.88 ± 0.70	0.64	**< 0.001**
		(0.50–0.93)		(0.57–1.29)		(0.50–1.21)	
Use of TV (†)	1.60 ± 081	1.29	1.48 ± 0.80	1.29	1.54 ± 0.81	1.29	0.099
		(1.00–2.00)		(0.93–1.86)		(0.93–2.00)	
Sedentary leisure (h/day) (†)	2.34 ± 1.08	2.21	2.48 ± 1.23	1.64	1.78 ± 0.98	2.21	0.303
		(1.71–2.79)		(1.21–2.29)		(1.64–3.14)	
**Adherence to recommendations**							
	***n*(%)**		***n*(%)**		***n*(%)**		***p***
Screen time ≤ 2 h/day	75 (42.9)		71 (41.0)		146 (41.9)		0.731
MVPA ≥60 min/day	42 (23.5)		76 (42.0)		118 (32.7)		**< 0.001**

*SD* Standard deviation; *IQR* Interquartile range; *PAL* Physical activity level; *MVPA* Moderate to vigorous physical activity.

Significant differences according to sex (*p* < 0.05) are marked as bold.

†: *p*-value calculated by Mann–Whitney U-test.

Furthermore, adherence to physical activity guidelines is also significantly higher in case of boys (42.0% vs. 23.46%; *p <* 0.001). However, males also spent more time using electronic displays such as PCs, tablets or videogame consoles (1.01 ± 0.78 h/day vs. 0.76 ± 0.58 h/day; *p <* 0.001).

Even though no significant differences among sexes were found related to adherence to sedentary guidelines, it is important to highlight that adherence to sedentary behavior recommendation was low (41.95%), with more than a half of the study population being sedentary. Furthermore, the percentage of the sample that adheres to physical activity guidelines is also below the half (32.7%), being lower the adherence of girls respect to boys (*p* < 0.001).

### Sports preferences by sex

**Fig. 2:**
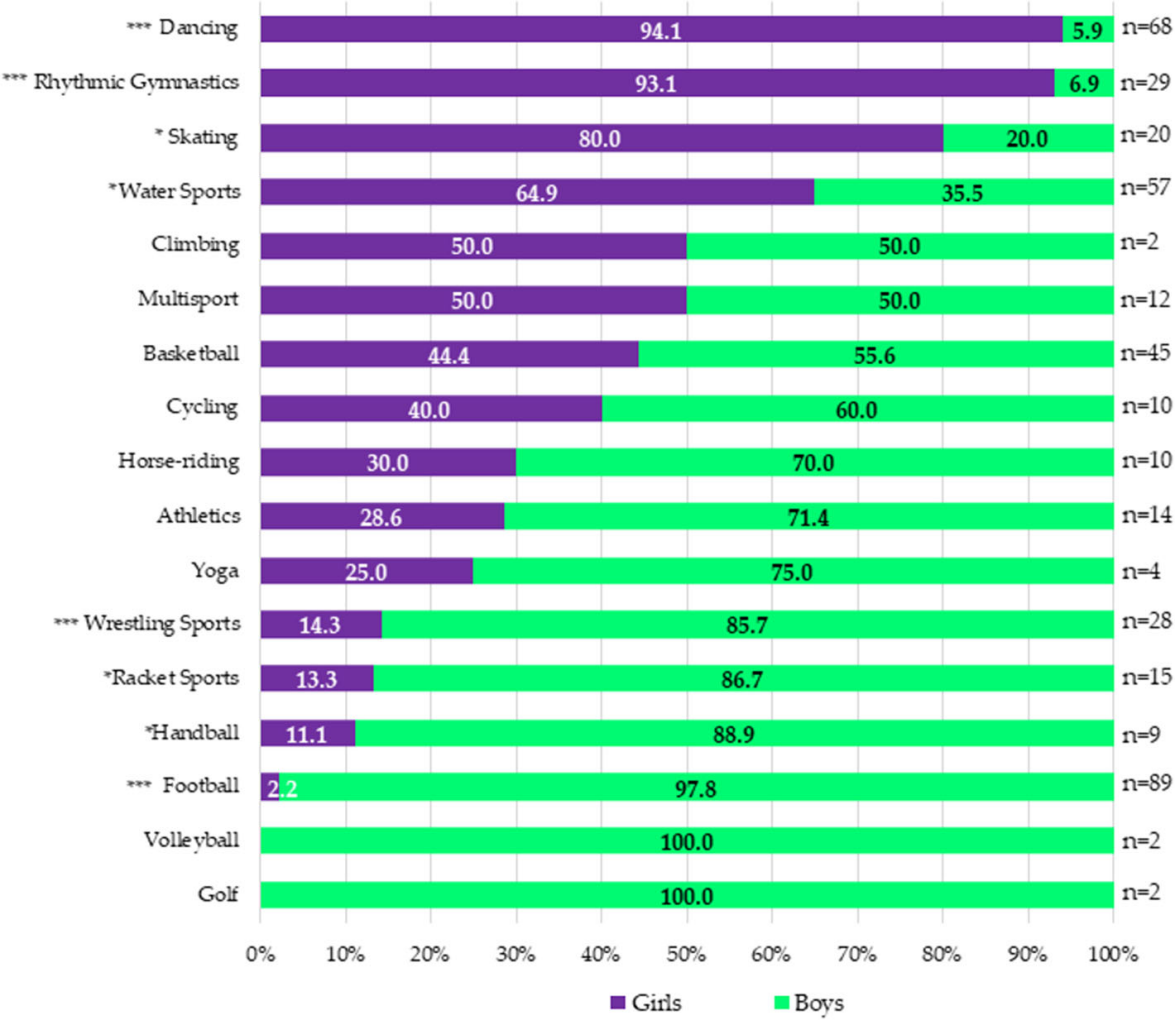
Sports preferences depending on sex. *p < 0.05 ***p < 0.001. a) *p*-value represents differences among sexes

Figure 2 shows that the most practiced sport was football (*n* = 89), followed by dancing (*n* = 68), water sports (*n* = 57), and basketball (*n* = 45). By contrast, the less practiced sports were golf, volleyball, and climbing (*n* = 2), followed by yoga (n = 4). Comparing preferences by sex, the results showed that girls were more inclined to choose dancing (*p < 0.001*), rhythm gymnastics (*p < 0.001*), skating (*p* = 0.005), and water sports (*p* = 0.012), while the boys opted for football (*p < 0.001*), wrestling sports (*p < 0.001*), racket sports (*p* = 0.004), and handball (*p* = 0.020). For the rest of sports, no significant differences were found among sexes.

The percentage of non-sedentary boys (PAL ≥1.4) was higher than the percentage of non-sedentary girls (82.9% vs. 68.2%; *p* = 0.001).

Table 3 shows the association between active children (PAL ≥1.4) and the sex of the caregiver. Children cared by their father were more likely to engage in physical activity (OR = 1.995 (1.202–3.310), *p* = 0.008), which was not seen in those cared by their mother. After adjusting for age, sex, and the rest of the predictor variables, this association was also observed in children cared by a person different from the father or the mother (OR = 2.222 (1.136–4.343), *p* = 0.020). However, it was not known if this person was a male or female figure.

**Table 3:** Odds ratios and 95% confidence intervals for the practice of physical activity (PAL≥1.4) depending on the person in charge of the child’s care

Predictor Variables		Model 1			Model 2			Model 3		
Questions	Groups	OR	CI 95%	***p***	OR	CI 95%	***p***	OR	CI 95%	***P***
The father takes care of the child (*n* = 185)	No	1		–	1		–	1		–
	Yes	**1.918**	**1.176–3.129**	**0.009**	**1.873**	**1.140–3.078**	**0.013**	**1.995**	**1.202–3.310**	**0.008**
The mother takes care of the child (*n* = 332)	No	1		–	1		–	1		–
	Yes	1.615	0.726–3.597	0.240	1.637	0.725–3.700	0.236	2.187	0.914–5.231	0.079
Other person takes care of the child (*n* = 89)	No	1		–	1		–	1		–
	Yes	1.420	0.783–2.575	0.249	1.718	0.929–3.176	0.084	**2.222**	**1.136–4.343**	**0.020**

Model 1: Not adjusted; Model 2: Adjusted by sex and age; Model 3: Adjusted by sex, age, and the other predictor variables. Significant differences according to sex (*p* < 0.05) are marked as bold.

## Discussion

28.02% of the total sample presented overweight and the 10.16% were obese, without significant differences among sexes. The percentage of overweight is higher than that in other studies carried out in the Spanish population, such as the ALADINO study, where in a sample of 10,899 children aged between 6 and 9 years, the percentage of overweight and obesity using the IOTF cut-off points was 21.8 and 11.2%, respectively [30]. This may be due to the prepubertal adipose rebound previously described by other authors [31, 32], as our sample includes children up to 11 years old.

In our results, a higher and more intense physical activity practice by boys than by girls was appreciable, being higher their PAL (1.46 ± 0.07 vs. 1.44 ± 0.07; *p <* 0.001) the time spent in MVPA (0.90 ± 0.45 h/day vs. 0.74 ± 0.40 h/day; *p <* 0.001) (Table 2) and their adherence to physical activity recommendations (42.0% vs. 23.46%; *p* = 0.000),as it occurred in other studies with schoolchildren population, such as the ANIBES study in Spain [33], the Youth Study in China [34] or the study of Williamson et al. performed in England and Scotland [35]. A possible explanation to this situation is that during school breaks or after-school day care, boys usually take advantage of this time to practice sports, while girls use this time in sedentary activities focused on socialization [36–38]. This is reflected, too, in the differences found among sexes when time dedicated to active play was analyzed, as boys spent more time on active play than girls (*p* = 0.014), which is partly attributable to the poor distribution of space in school playgrounds or sports facilities, as it does not take into account different sporting preferences, to the prejudice of those children who do not use material like football goal posts or basketball nets, who are generally girls [37, 39]. Related to these findings, we saw that boys also attended extracurricular sport classes more frequently and for more hours than girls (2.7 ± 1.6 days/week vs. 1.8 ± 1.4 days/week; *p < 0.001* and 0.50 ± 0.36 h/day vs. 0.38 ± 0.34 h/day; *p* = 0.001, respectively) (Table 2). These kinds of differences are similar to those observed in the ALADINO study [30], where boys also spent more time in the practice of extracurricular sport activities than girls.

The mean time spent in sedentary leisure activities was 2.34 ± 1.08 h by girls and 2.48 ± 1.23 h by boys, being found significant differences between sexes only when time spent using PCs, tablets or game stations was analyzed, being higher the time dedicated by boys to this kind of activity (*p* < 0.001). Other studies showed this higher time spent by boys in recreational use of computers or other electronic devices [40], even though girls usually spent more time in sedentary behaviors [41].

Regarding sports preferences collected in Fig. 2, the data showed that children tend to choose activities in line with their gender roles, with a higher female participation in sports socially perceived as feminine, like dancing (94.1% girls vs. 5.9% boys; *p* < 0.001) or rhythmic gymnastics (93.1% girls vs. 6.9% boys; *p* < 0.001), while males participated more in sports socially perceived as masculine, like football (97.8% boys vs. 2.2% girls; *p* < 0.001) or wrestling sports (85.7% boys vs. 14.3% girls; *p* < 0.001) [42, 43]. Furthermore, girls also participated more than boys in skating (80% girls vs. 20% boys; *p* = 0.005) and water sports (64.9% girls vs. 35.1% boys; *p* = 0.012), and boys enrolled more in handball (88.9% boys vs. 11.1% girls; *p* = 0.02) and racket sports (86.7% boys vs. 13.3% girls; *p* = 0.004), which is in line with the literature previously mentioned, where a greater tendency of boys to practice team sports was reflected in contrast to girls, who tended to practice individual ones [10, 11, 44]. These results are similar to those found in other studies that analyzed children’s sports preferences [16, 45, 46] and seem to remain true throughout life [47]. Sports segregation according to gender roles may be related to the fear of being judged or bullied if gender norms are not conformed to [37, 48]. In fact, various articles have shown that girls are more likely to engage in team sports when other girls are playing but not when boys are playing, because boys may exclude girls when they try to participate in sports in which girls are not socially considered good enough [49–51].

Another relevant aspect of this research is the association between parental care and the practice of physical activity by the child shown in Table 3. In relation to this, children are significantly at higher odds of engaging in physical activity when the father takes care of them (*p* = 0.008), an association not seen in mother care. Nevertheless, disparities have been found in the literature in this respect. For example, the results described by Rodrigues et al. [15], as well as those described by Fuemmeler et al. [52], showed that children were more likely to engage in physical activity if both parents or the parent who shared sex with them was active, whereas other studies revealed results more similar to those found in our research, showing a greater relationship between the physical activity of the child and the influence of the father [53, 54]. This fact reinforces the theory that physical activity is culturally assumed as a masculine domain. However, it is also important to highlight the positive association with physical activity practice by children when a person different from their parents habitually takes care of them (*p* = 0.020). Although this finding differs from the previous literature [55], it can be related to time disposal by the person in charge of the care of the child, which is exploited to spend time with the child accompanying them to the park or in sports practice [56].

### Strengths and limitations

The current study is one of the few to analyze the different sports preferences and parental influence on children physical activity from a gender perspective. However, there are some limitations. One of these limitations is the cross-sectional design, which does not allow us to make causal inferences on the observed associations. As the study could not be carried out in all the Autonomous Communities needed, our sample is not a representative sample of the Spanish population, so the results are not representative of all the Spanish schoolchildren and they are not applicable to other age groups. Due to this low participation rate, the sample could be biased to some extent with families specially interested in their children’s health, which can imply higher physical activity rates than in general population. Also, this is a secondary analysis from the project “Sodium Sources and Sodium Intake in a Representative Sample of Spanish Children”, which was focused on evaluating the sodium intake of Spanish schoolchildren through 24-h urine samples. The difficulty in the collection of this measure has affected the participation in the study, so the final sample was conformed to the 20.15% of the contacted children. Furthermore, we just had the possibility to use questionnaires to collect physical activity, instead of another more objective method as accelerometer, which may lead to a bias towards underestimation or overestimation of the physical activity practice by schoolchildren. Therefore, the true associations could have been stronger or weaker than the observed associations, depending on whether the misclassification was differential or non-differential.

## Conclusions

The findings of this study indicated that girls practice less physical activity and less intensely than boys, engaging more in individual sports with artistic connotations, while boys engage more in team sports or sports with a high physical contact component.

On the other hand, when the father is in charge or takes care of the child, it is more probable that the child will be more physically active, independently of whether or not the mother is also involved in his or her care.

Considering all the above, the creation of gender policies which take into account differences in sports preferences could foster the practice of physical activity by children, especially girls, who are actually less favored in this aspect.

However, other longitudinal or intervention studies should be carried out to analyze whether these differences in sports practice between boys and girls can lead to differences in the health status of children.

## Supplementary information

**Additional file 1.** Socio-sanitary questionnaire. Questionnaire used to obtain data on the children’s caregivers, including the academic level of their parents and the household incomes.

**Additional file 2.** Daily physical activity questionnaire. Questionnaire used to obtain data on children’s physical activity, sedentary behavior and extracurricular sport classes.

## Data Availability

The datasets generated and an alysed during the current study are not publicly available due to ethical restrictions and participant confidentiality but are available from the corresponding author on reasonable request.

## Abbreviations

BMI: Body mass index
CI: Confidence interval
IOM: Institute of Medicine
IQR: Interquartile range
MVPA: Moderate to vigorous physical activity
NAM: National Academy of Medicine
OR: Odds ratio
PAL: Physical activity level
SD: Standard deviation
VT: Vocational training
WHO: World Health Organization
