# Supplementary material for: Physical activity practice and sports preferences in a group of Spanish schoolchildren depending on sex and parental care: a gender perspective
Source: BMC Pediatr. 2020 Jul 7;20:337. doi: 10.1186/s12887-020-02229-z (PMC7339494; doi:10.1186/s12887-020-02229-z)
Supplement: Supplementary file 1 — Additional file 1. Socio-sanitary questionnaire. Questionnaire used to obtain data on the children’s caregivers, including the academic level of their parents and the household incomes. [file 12887_2020_2229_MOESM1_ESM.docx]

CUESTIONARIO SOCIO-SANITARIO

(A rellenar por los padres/madres/tutores)

1. Persona que rellena el cuestionario:
   - Madre
   - Padre
   - Otro Especificar: ………................
2. Nombre y apellidos del niño/a: _________________________________________

Teléfono/móvil: Correo electrónico:

Dirección postal:

1. Fecha de nacimiento del niño/a: │__│__│Día │__│__│Mes │__│__│__│__│Año
2. ¿Cuál es el lugar de nacimiento del niño/a y de usted y de su pareja?

|  | **NIÑO/A** | **PADRE** | **MADRE** |
| --- | --- | --- | --- |
| España |  |  |  |
| Otro país miembro de la UE |  |  |  |
| Otro país no UE |  |  |  |

1. En caso de no haber nacido en España, indique la nacionalidad y el tiempo que lleva residiendo en España:

|  | **NIÑO/A** | **PADRE** | **MADRE** |
| --- | --- | --- | --- |
| Nacionalidad |  |  |  |
| Tiempo de residencia en España |  |  |  |

1. Incluyendo a su hijo/a, indique las personas que conviven en el domicilio familiar:
   - Nº de personas ≥ 18 años: │__│__│
   - Nº de personas < 18 años: │__│__│
2. ¿Cuál es el nivel de estudios más alto que ha realizado usted y su pareja?

| **PADRE** | | **MADRE** | |
| --- | --- | --- | --- |
| Sin estudios |  | Sin estudios |  |
| Primaria |  | Primaria |  |
| Secundaria/FP |  | Secundaria/FP |  |
| Diplomatura/Licenciatura |  | Diplomatura/Licenciatura |  |
| Máster/Doctorado |  | Máster/Doctorado |  |

1. ¿Cuál fue el nivel de ingresos brutos en su hogar el año pasado?

| **INGRESOS** |  |
| --- | --- |
| Menos de 12.000 € |  |
| Entre 12.000€ y 18.000€ |  |
| Entre 18.001€ y 24.000€ |  |
| Entre 24.001€ y 30.000€ |  |
| Entre 30.001€ y 36.000€ |  |
| Entre 36.001€ y 42.000€ |  |
| Entre 42.001€ y 48.000€ |  |
| Más de 48.000€ |  |
| NS/NC |  |

1. ¿Cuál de las siguientes respuestas describe su situación laboral y la de su pareja?

| **PADRE** | | **MADRE** | |
| --- | --- | --- | --- |
| Funcionario público |  | Funcionario público |  |
| Empresa privada |  | Empresa privada |  |
| Autónomo |  | Autónomo |  |
| Estudiante |  | Estudiante |  |
| Labores del hogar |  | Labores del hogar |  |
| Desempleado capacitado para trabajar |  | Desempleado capacitado para trabajar |  |
| Desempleado incapacitado para trabajar |  | Desempleado incapacitado para trabajar |  |
| Jubilado |  | Jubilado |  |

1. Por favor, rellene los siguientes datos:

| **PADRE** | | **MADRE** | |  |
| --- | --- | --- | --- | --- |
| Edad: | | Edad: | |  |
| Peso: | | Peso: | |  |
| Altura: | | Altura: | |  |
| ¿Fuma? Sí No  (Si la respuesta es sí indique el número de cigarros/día): | | ¿Fuma? Sí No  (Si la respuesta es sí indique el número de cigarros/día): | |  |
| ¿Dónde fuma? (casa, trabajo, etc): | | ¿Dónde fuma? (casa, trabajo, etc): | |  |
| **Enfermedades** | | **Especificar si usted o su pareja padece alguna de las enfermedades mencionadas** | | |
|  |  | **PADRE** | | **MADRE** |
| Colesterol elevado | | Sí No NS/NC | | Sí No NS/NC |
| Hipertensión | | Sí No NS/NC | | Sí No NS/NC |
| Diabetes | | Sí No NS/NC | | Sí No NS/NC |
| Osteoporosis | | Sí No NS/NC | | Sí No NS/NC |
| Obesidad | | Sí No NS/NC | | Sí No NS/NC |
| Otras enfermedades (especificar): | | Sí No NS/NC | | Sí No NS/NC |

1. Indique el peso del niño o niña al nacer: │__│,│__│__│__│(kg)
2. ¿Siguió lactancia materna?
   - Sí En caso afirmativo indicar meses: │__│__│
   - No
3. ¿Su hijo/a tiene alguna dificultad de aprendizaje (problemas o dificultades de atención, de lectura y/o escritura, de cálculo o matemáticas…)?
   - Sí
   - No

Si su respuesta anterior fue SÍ tiene dificultad, por favor especifique aquí cuál:

………………………………………………………………………………………………………………………..

1. ¿En la actualidad está consultando a algún profesional por el/los problemas que ha señalado antes?
   - Sí
   - No

¿Con qué profesional (psicólogo, logopeda…)? ………………………………………………………

1. Indique si su hijo padece alguna enfermedad y/o alergia:

| **Enfermedades** | **Especificar si su hijo padece alguna de las enfermedades mencionadas** |
| --- | --- |
| Colesterol elevado | Sí No NS/NC |
| Hipertensión | Sí No NS/NC |
| Diabetes | Sí No NS/NC |
| Obesidad | Sí No NS/NC |
| Asma | Sí No NS/NC |
| Otras enfermedades (especificar): | Sí No NS/NC |
| Alergia alimentaria (especificar): | Sí No NS/NC |
| Otras alergias (especificar): | Sí No NS/NC |

1. Anote si su hijo/a ha tomado en el último mes algún tipo de medicamento (gotas, pastillas, inyecciones, supositorios, pomadas, etc):

| **Nombre** | **Dosis** |
| --- | --- |
|  |  |
|  |  |

1. Anote si su hijo/a ha tomado en el último mes algún tipo de suplemento de vitaminas y/o minerales u otros complementos:

| **Nombre** | **Dosis** |
| --- | --- |
|  |  |
|  |  |

1. Usted considera que el peso de su hijo/a es:
   - Insuficiente
   - Adecuado
   - Excesivo
2. De acuerdo con su peso actual, le gustaría que su hijo/a pesara:
   - Menos
   - Igual
   - Más
3. ¿Está siguiendo su hijo/a algún tipo de dieta?
   - Sí (por enfermedad)
   - Sí (de adelgazamiento)
   - Sí (otra causa) especificar:
   - No
4. ¿Quién se encarga de la compra de los alimentos?
   - Madre
   - Padre
   - Otros (especificar):
5. ¿Quién se encarga de la preparación de las comidas del niño/a?
   - Madre
   - Padre
   - Otros (especificar):
6. ¿Quién se encarga del niño/a cuando no está en el colegio?
   - Madre
   - Padre
   - Otros (especificar):

SOCIO-SANITARY QUESTIONNAIRE (English version)

(To fill out by parents/guardians)

1. Person who fills out the questionnaire:
   - Mother
   - Father
   - Other Specify: ………................
2. Name and surname of the child: ________________________________________

Phone number: E-mail:

Postal address:

1. Date of birth of the child: │__│__│Day │__│__│Month │__│__│__│__│Year
2. What is the birthplace of the child and of the mother and father?

|  | **CHILD** | **FATHER** | **MOTHER** |
| --- | --- | --- | --- |
| Spain |  |  |  |
| Other country from the EU |  |  |  |
| Other non-EU country |  |  |  |

1. If you were not born in Spain, please indicate your nationality and how long you have been residing in Spain:

|  | **CHILD** | **FATHER** | **MOTHER** |
| --- | --- | --- | --- |
| Nacionality |  |  |  |
| Length of residence in Spain |  |  |  |

1. Including your child, indicate the people living in the family home:
   1. Nº of people ≥ 18 years old: │__│__│
   2. Nº of people < 18 years old: │__│__│
2. What is the highest level of education completed by both parents?

| **FATHER** | | **MOTHER** | |
| --- | --- | --- | --- |
| No academic education |  | No academic education |  |
| Primary school |  | Primary school |  |
| High school/VT |  | High school/VT |  |
| University degree |  | University degree |  |
| Master/PhD |  | Master/PhD |  |

1. What was the gross income level in your household last year?

| **ICOME** | |
| --- | --- |
| Less than 12,000 € |  |
| Between 12,000€ and 18,000€ |  |
| Between 18,001€ and 24,000€ |  |
| Between 24,001€ and 30,000€ |  |
| Between 30,001€ and 36,000€ |  |
| Between 36,001€ and 42,000€ |  |
| Between 42,001€ and 48,000€ |  |
| More than 48,000€ |  |
| DK/DA |  |

1. Which of the following answers describes the work situation of the mother and father?

| **FATHER** | | **MOTHER** | |
| --- | --- | --- | --- |
| Civil servant |  | Civil servant |  |
| Private company |  | Private company |  |
| Self-employed |  | Self-employed |  |
| Student |  | Student |  |
| Household chores |  | Household chores |  |
| Unemployed able to work |  | Unemployed able to work |  |
| Unemployed unable to work |  | Unemployed unable to work |  |
| Retired |  | Retired |  |

1. Please fill in the following data:

| **FATHER** | | **MOTHER** | |  |
| --- | --- | --- | --- | --- |
| Age: | | Age: | |  |
| Weight: | | Weight: | |  |
| Height: | | Height: | |  |
| ¿Do you smoke? Yes No  (If answer is yes, indicate the number of cigarettes per day: | | ¿Do you smoke? Yes No  (If answer is yes, indicate the number of cigarettes per day: | |  |
| ¿Where do you smoke? (at home, at work, etc.): | | ¿Where do you smoke? (at home, at work, etc.): | |  |
| **DISEASE** | | **Specify if the father or mother suffers from any of the above diseases** | | |
|  |  | **FATHER** | | **MOTHER** |
| High cholesterol | | Yes No DK/DA | | Yes No DK/DA |
| Hypertension | | Yes No DK/DA | | Yes No DK/DA |
| Diabetes | | Yes No DK/DA | | Yes No DK/DA |
| Osteoporosis | | Yes No DK/DA | | Yes No DK/DA |
| Obesity | | Yes No DK/DA | | Yes No DK/DA |
| Other diseases (specify): | | Yes No DK/DA | | Yes No DK/DA |

1. Indicate the weight of the child at birth: │__│.│__│__│__│(kg)
2. Did the child breast-feed?
   1. Yes If yes, please indicate months: │__│__│
   2. No
3. Your child has any learning difficulty (problems or difficulties with attention, reading and/or writing, calculus or mathematics...)?
   1. Yes
   2. No

If the previous answer was YES she/he has a difficulty, please specify here which:

………………………………………………………………………………………………………………………..

1. Is your child currently consulting a professional for the problem(s) you have identified above?
   1. Yes
   2. No

What kind of professional (psychologist, speech therapist…)? ………………………………………………………

1. Indicate if your child has any illnesses and/or allergies:

| **DISEASES** | **Specify if your child has any of the diseases listed above** |
| --- | --- |
| High cholesterol | Yes No DK/DA |
| Hypertension | Yes No DK/DA |
| Diabetes | Yes No DK/DA |
| Obesity | Yes No DK/DA |
| Asthma | Yes No DK/DA |
| Other diseases (specify): | Yes No DK/DA |
| Food allergy (specify): | Yes No DK/DA |
| Other allergies (specify): | Yes No DK/DA |

1. Record whether your child has taken any medications (drops, tablets, injections, suppositories, ointments, etc.) in the past month:

| **Name** | **Dose** |
| --- | --- |
|  |  |
|  |  |

1. Record whether your child has taken any vitamin and/or mineral supplements or other supplements in the past month:

| **Name** | **Dose** |
| --- | --- |
|  |  |
|  |  |

1. You consider the weight of your child:
   1. Insufficient
   2. Adequate
   3. Excessive
2. Based on his or her current weight, you would like your child to weigh:
   1. Less
   2. Equal
   3. More
3. Is your child following any kind of diet?
   1. Yes (due to a disease)
   2. Yes (to lose weight)
   3. Yes (other cause) specify:
   4. No
4. Who is in charge of buying the food?
   1. Mother
   2. Father
   3. Other (specify):
5. Who is in charge of preparing the meals for the child?
   1. Mother
   2. Father
   3. Other (specify):
6. Who takes care of the child when he or she is not in school?
   1. Mother
   2. Father
   3. Other (specify):
