# Supplementary material for: Physical activity practice and sports preferences in a group of Spanish schoolchildren depending on sex and parental care: a gender perspective
Source: BMC Pediatr. 2020 Jul 7;20:337. doi: 10.1186/s12887-020-02229-z (PMC7339494; doi:10.1186/s12887-020-02229-z)
Supplement: Supplementary file 2 — Additional file 2. Daily physical activity questionnaire. Questionnaire used to obtain data on children’s physical activity, sedentary behavior and extracurricular sport classes. [file 12887_2020_2229_MOESM2_ESM.docx]

CUESTIONARIO DE ACTIVIDAD FÍSICA

(A rellenar por los padres/madres/tutores)

Nombre y apellidos del niño/a: _______________________________________________________

Persona que rellena el cuestionario: Madre □ Padre □ Otro (especificar) □: __________________

**Indique el tiempo (horas o minutos) empleados en la realización de cada actividad, de forma que el tiempo total de cada una de las columnas sume 24 horas.**

| **Actividad** | **Día laborable (promedio de 5 días)** | **Fines de semana (promedio de 2 días)** |
| --- | --- | --- |
| Dormir (incluir siestas) |  |  |
| Actividades que se realizan sentado (estudiar, hablar con amigos, etc.) |  |  |
| Comer (incluir todas las comidas realizadas en el día) |  |  |
| Actividades que se realizan de pie (conversar, esperar, etc.) |  |  |
| Pasear, andar, desplazamientos |  |  |
| Jugar de forma activa (en la calle, parques, recreo, etc.) |  |  |
| Educación física realizada en el colegio |  |  |
| Clases extraescolares deportivas (fútbol, baile, baloncesto, natación, etc.) |  |  |
| Ocio sedentario (televisión, videojuegos, tablet, móvil con internet, etc.) |  |  |
| Otros (especificar): |  |  |

1. ¿A qué hora se levanta y se acuesta su niño/a?

|  | **Días laborables** | **Fines de semana** |
| --- | --- | --- |
| Hora a la que se levanta: | \|__\|__\| Horas \|__\|__\| Minutos | \|__\|__\| Horas \|__\|__\| Minutos |
| Hora a la que se acuesta: | \|__\|__\| Horas \|__\|__\| Minutos | \|__\|__\| Horas \|__\|__\| Minutos |

1. En una semana normal, indique las **actividades extraescolares deportivas** que realiza su niño/a en cada día de la semana e indique el tiempo empleado en cada actividad por sesión:

| Día de la semana | Actividades extraescolares **deportivas** (especificar) | Tiempo empleado **por sesión o día de entrenamiento** |
| --- | --- | --- |
| L |  | \|__\|__\| Horas \|__\|__\| Minutos  \|__\|__\| Horas \|__\|__\| Minutos |
| M |  | \|__\|__\| Horas \|__\|__\| Minutos  \|__\|__\| Horas \|__\|__\| Minutos |
| X |  | \|__\|__\| Horas \|__\|__\| Minutos  \|__\|__\| Horas \|__\|__\| Minutos |
| J |  | \|__\|__\| Horas \|__\|__\| Minutos  \|__\|__\| Horas \|__\|__\| Minutos |
| V |  | \|__\|__\| Horas \|__\|__\| Minutos  \|__\|__\| Horas \|__\|__\| Minutos |
| S |  | \|__\|__\| Horas \|__\|__\| Minutos  \|__\|__\| Horas \|__\|__\| Minutos |
| D |  | \|__\|__\| Horas \|__\|__\| Minutos  \|__\|__\| Horas \|__\|__\| Minutos |

1. Indique qué dispositivos están a disposición de su hijo/a en el hogar:

| Ordenador/Tablet |  |
| --- | --- |
| Videoconsola |  |
| Teléfono con internet |  |
| Televisión en la habitación del niño/a |  |

1. ¿Cuántas horas al día dedica habitualmente su niño/a a usar el ordenador, o consolas de videojuegos, o dispositivos similares para jugar, ya sea en casa o en otro lugar, en su tiempo libre? Responder tanto para los días entre semana como para los fines de semana.

| **ENTRE SEMANA** | | **FINES DE SEMANA** | |
| --- | --- | --- | --- |
| Ninguna |  | Ninguna |  |
| Menos de 1 hora al día |  | Menos de 1 hora al día |  |
| Alrededor de 1 horas al día |  | Alrededor de 1 horas al día |  |
| Alrededor de 2 horas al día |  | Alrededor de 2 horas al día |  |
| Alrededor de 3 horas al día |  | Alrededor de 3 horas al día |  |
| Alrededor de 4 horas al día |  | Alrededor de 4 horas al día |  |
| Alrededor de 5 horas al día |  | Alrededor de 5 horas al día |  |
| 6 o más horas al día |  | 6 o más horas al día |  |

1. ¿Cuántas horas al día dedica habitualmente su niño/a a ver la televisión (incluidos vídeos, DVD, etc.), ya sea en casa o en otro lugar, en su tiempo libre? Responder tanto para los días entre semana como para los fines de semana.

| **ENTRE SEMANA** | | **FINES DE SEMANA** | |
| --- | --- | --- | --- |
| Ninguna |  | Ninguna |  |
| Menos de 1 hora al día |  | Menos de 1 hora al día |  |
| Alrededor de 1 horas al día |  | Alrededor de 1 horas al día |  |
| Alrededor de 2 horas al día |  | Alrededor de 2 horas al día |  |
| Alrededor de 3 horas al día |  | Alrededor de 3 horas al día |  |
| Alrededor de 4 horas al día |  | Alrededor de 4 horas al día |  |
| Alrededor de 5 horas al día |  | Alrededor de 5 horas al día |  |
| 6 o más horas al día |  | 6 o más horas al día |  |

PHYSICAL ACTIVITY QUESTIONNAIRE (English version)

(To fill out by parents/guardians)

Name and surname of the child: ______________________________________________________

Person who fills out the questionnaire: Mother □ Father □ Other (specify) □: _________________

**Indicate the time (hours or minutes) used in carrying out each activity, so that the total time of each of the columns reaches 24 hours.**

| **Activity** | **Weekday**  **(Average 5 days)** | **Weekend**  **(Average 2 days)** |
| --- | --- | --- |
| Sleep (including naps) |  |  |
| Sitting activities (studying, talking to friends, etc.) |  |  |
| Eating (including all meals during the day) |  |  |
| Activities that are performed standing (talking, waiting, etc.). |  |  |
| Walking, hiking, displacements |  |  |
| Active play (in the street, parks, recreation, etc.) |  |  |
| Physical education at school |  |  |
| After-school sports classes (football, dance, basketball, swimming, etc.) |  |  |
| Sedentary leisure (television, video games, tablet, mobile with internet, etc.). |  |  |
| Other (specify): |  |  |

1. What time does your child wake up and go to bed?

|  | **Weekdays** | **Weekends** |
| --- | --- | --- |
| Wake up time: | \|__\|__\| Hours \|__\|__\| Minutes | \|__\|__\| Hours \|__\|__\| Minutes |
| Bedtime: | \|__\|__\| Hours \|__\|__\| Minutes | \|__\|__\| Hours \|__\|__\| Minutes |

1. In a typical week, list the **extracurricular sports activities** your child practices on each day of the week and the time spent on each activity per session:

| Day of the week | Extracurricular **sport** classes (specify) | Time spent **per session or day of training** |
| --- | --- | --- |
| Mon |  | \|__\|__\| Hours \|__\|__\| Minutes  \|__\|__\| Hours \|__\|__\| Minutes |
| Tue |  | \|__\|__\| Hours \|__\|__\| Minutes  \|__\|__\| Hours \|__\|__\| Minutes |
| Wed |  | \|__\|__\| Hours \|__\|__\| Minutes  \|__\|__\| Hours \|__\|__\| Minutes |
| Thu |  | \|__\|__\| Hours \|__\|__\| Minutes  \|__\|__\| Hours \|__\|__\| Minutes |
| Fri |  | \|__\|__\| Hours \|__\|__\| Minutes  \|__\|__\| Hours \|__\|__\| Minutes |
| Sat |  | \|__\|__\| Hours \|__\|__\| Minutes  \|__\|__\| Hours \|__\|__\| Minutes |
| Sun |  | \|__\|__\| Hours \|__\|__\| Minutes  \|__\|__\| Hours \|__\|__\| Minutes |

1. Indicate what devices are available to your child at home:

| Computer/Tablet |  |
| --- | --- |
| Videogame |  |
| Mobile phone with internet |  |
| TV in the room of the child |  |

1. How many hours per day does your child usually use the computer, or video game consoles, or similar devices to play, either at home or elsewhere, in his/her free time? Respond to both weekdays and weekends.

| **WEEKDAYS** | | **WEEKENDS** | |
| --- | --- | --- | --- |
| None |  | None |  |
| Less than 1 hour per day |  | Less than 1 hour per day |  |
| About 1 hour a day |  | About 1 hour a day |  |
| About 2 hours a day |  | About 2 hours a day |  |
| About 3 hours a day |  | About 3 hours a day |  |
| About 4 hours a day |  | About 4 hours a day |  |
| About 5 hours a day |  | About 5 hours a day |  |
| 6 or more hours per day |  | 6 or more hours per day |  |

1. How many hours per day does your child usually watch television (including videos, DVDs, etc.), either at home or elsewhere, in his/her free time? Respond both on weekdays and weekends.

| **WEEKDAYS** | | **WEEKENDS** | |
| --- | --- | --- | --- |
| None |  | None |  |
| Less than 1 hour per day |  | Less than 1 hour per day |  |
| About 1 hour a day |  | About 1 hour a day |  |
| About 2 hours a day |  | About 2 hours a day |  |
| About 3 hours a day |  | About 3 hours a day |  |
| About 4 hours a day |  | About 4 hours a day |  |
| About 5 hours a day |  | About 5 hours a day |  |
| 6 or more hours per day |  | 6 or more hours per day |  |
